# The effect of radiation therapy in the treatment of adult soft tissue sarcomas of the extremities: a long‐term community‐based cancer center experience

**DOI:** 10.1002/cam4.972

**Published:** 2017-02-11

**Authors:** Jeffrey S. Kneisl, Chad Ferguson, Myra Robinson, Anthony Crimaldi, Will Ahrens, James Symanowski, Michael Bates, Jennifer L. Ersek, Michael Livingston, Joshua Patt, Edward S. Kim

**Affiliations:** ^1^ Department of Orthopedic OncologyLevine Cancer InstituteCarolinas HealthCare SystemCharlotteNorth Carolina; ^2^ Department of Cancer BiostatisticsLevine Cancer InstituteCarolinas HealthCare SystemCharlotteNorth Carolina; ^3^ Department of Radiation OncologyLevine Cancer InstituteCarolinas HealthCare SystemCharlotteNorth Carolina; ^4^ Department of PathologyCarolinas Pathology GroupCharlotteNorth Carolina; ^5^ Department of Solid Tumor Oncology and Investigational TherapeuticsLevine Cancer InstituteCarolinas HealthCare SystemCharlotteNorth Carolina

**Keywords:** Adult, complications, extremities, radiotherapy, sarcoma, survival

## Abstract

The aim of the study was to determine the effect of external beam radiotherapy (RT) in the treatment of extremity soft tissue sarcoma (STS) before or after limb‐sparing surgery (LSS) in a community‐based setting. Patients presenting to our institution from 1992 to 2010 and meeting eligibility criteria were stratified into low (G1) or high (G2, G3) pathologic grade and evaluated. Major complication events, including amputation, radiation‐induced sarcoma, and pathologic fracture, were assessed. Kaplan–Meier techniques and Cox proportional hazards regression models were used. One hundred and sixty‐two eligible patients underwent LSS for extremity STS (120 high grade, 42 low grade). Median time of follow‐up was 5.1 years (0.8–20.3 years). RT was administered to 111 patients. In unadjusted models, RT significantly decreased the risk of local recurrence (LR) in high‐grade STS patients (*P* = 0.005) and had a trend for improved recurrence‐free survival (RFS) (*P* = 0.069). In multivariable‐adjusted models, RT significantly improved time to LR (*P* = 0.001), RFS (*P* = 0.003), and overall survival (OS) (*P* = 0.003). Analysis of all patients showed those who underwent RT had a major complication rate (MCR) of 16.2%, compared to 3.9% in the no RT group (*P* = 0.037); however, the difference in MCR did not differ significantly when the analysis was restricted to high‐grade sarcomas. In our large experience of patients with extremity STS undergoing limb sparing surgery (LSS), RT significantly improved local recurrence (LR), RFS, and OS, in patients with high‐grade tumors. Efficacy benefits of RT should be weighed against potential complications. External beam RT should be considered in patients with resected high‐grade sarcomas.

## Introduction

In the United States, over 15,000 people are diagnosed with sarcomas annually with 6200 deaths with the majority being soft tissue sarcomas (STS)^1^. A multidisciplinary approach includes limb sparing surgery (LSS), which is the standard treatment for adult STS of the extremities with goals of achieving a complete tumor resection, maximizing preservation of limb function, and minimizing morbidities. Chemotherapy (e.g., ifosfamide and doxorubicin) is used with radiation therapy (RT) to reduce recurrence or in the palliative setting. RT has been used since the 1960s 2, 3, both in the preoperative or postoperative setting 4. The National Comprehensive Cancer Network (NCCN) guidelines have postulated best treatment practices based on available literature; however, treatment approaches are not consistently standardized between academic and nonacademic centers 5, 6. Limited information is available on the impact of RT on overall survival (OS) and long‐term major complications 7, 8.

Two randomized clinical trials (RCTs) performed at NCI Comprehensive Cancer Centers comparing LSS versus LSS with postoperative radiation in patients with extremity STSs demonstrated a 20–25% reduction in the risk of LR with radiation 9, 10. Additional studies have reported RT in the neoadjuvant and adjuvant settings 11, 12 with no reported differences in local control, progression‐free survival (PFS), or OS. Mixed results regarding the use of radiation for improvement in disease‐specific survival and/or OS have been reported 9, 13, 14, 15, 16, 17, 18, 19. The purpose of this study, performed at a community‐based cancer center, was to determine the effect of RT on OS, recurrence‐free survival (RFS), local recurrence (LR), systemic recurrence, and to report the associated major complication rates (MCR), including secondary sarcomas, pathologic fractures, and amputation, in patients with STS.

## Methods

We conducted an IRB‐approved retrospective review of all patients diagnosed with extremity STS presenting to our institution from 1992 to 2010. Treatment course was decided prospectively at our multidisciplinary sarcoma tumor board. Eligible patients included as follows: diagnosis of a primary extremity STS, LSS, age 18+ years at presentation, no metastatic disease at presentation, and 2+ years of follow‐up. Patients with <2 years of follow‐up were included only if they experienced an event such as death, recurrence, or complication. Exclusion criteria included as follows: prior chemotherapy, histology of desmoid tumor, or dermatofibrosarcoma protuberans. Tumors were classified into low (G1) or high (G2/G3) pathologic grade. Patients were selected for RT (either 2D RT, 3D conformal RT, or IMRT techniques) according to physician decisions based on multidisciplinary discussion. RT dose was recorded. Major complications included amputation, radiation‐induced secondary sarcoma, and pathologic fracture. After index LSS, amputations were conducted for recurrence, infection, or chronic pain. Pathologic fractures were defined as fractures occurring in the radiation field post‐RT. Radiation‐induced sarcoma was defined as occurrence of a new malignancy within the irradiated field after successful index LSS with histology discordant with original sarcoma.

### Time to event endpoint definitions

OS was measured from date of LSS to date of death from any cause; surviving patients were censored at the date of last follow‐up. RFS was measured from the date of limb sparing surgery to date of recurrence (local or systemic) or death, whichever occurred first. Surviving patients who did not experience a recurrence were censored at the date of last follow‐up. Time to LR was measured from the date of LSS to the date of LR. Patients were censored at the date of systemic recurrence if they had a systemic recurrence, date of death if they died prior to experiencing a LR, or were censored at the date of last follow‐up. Time to systemic recurrence was measured from date of LSS to the date of systemic recurrence. Patients were censored at the date of death if they died prior to experiencing a systemic recurrence or at the date of last follow‐up. Local RFS was measured similarly to RFS, however, a local RFS event occurred only if the patient experienced a LR or death from any cause; subjects who experienced a systemic recurrence before a LR or death, or who were recurrence‐free and alive at the end of follow‐up were censored at the date of systemic recurrence or last follow‐up, respectively. Systemic RFS was also measured similarly to RFS, however, a systemic RFS event occurred only if the patient experienced a systemic recurrence or death from any cause; subjects were only censored if they did not experience a systemic recurrence or death before the end of follow‐up.

### Statistical analysis

Analyses were conducted for the entire cohort and subgroups of patients with high‐ and low‐grade histology. Frequency and proportion of patient and tumor characteristics were summarized overall and by RT group. Kaplan–Meier techniques were used to estimate survival distributions for each outcome and log‐rank tests compared the survival distributions between the RT groups. Cox proportional hazards regression was used to determine hazard ratios (HR) and 95% confidence intervals (CI) to estimate the magnitude of the impact of RT on survival outcomes. Univariate and multivariable Cox regression assessed the impact of patient and disease characteristics on survival outcomes and were used for estimation of adjusted hazard ratios. Multivariable Cox models were determined for each outcome using backward elimination and forward selection modeling procedures (significance levels of *P* = 0.10). Individual prognostic factors were identified through univariate Cox models for all potential covariates (gender, age, margin status, tumor size, tumor site, and tumor side). Significance of the adjusted RT hazard ratios were assessed at the *α *= 0.05 significance level.

Complication rates were compared between the treatment groups using Fisher's exact tests. Among those receiving RT, radiation dose was compared between high‐ and low‐grade STSs using a nonparametric rank test. Additionally, ANOVA models, utilizing a log transformation on the dose, were used to compare radiation dose between margin status groups in patients with adjuvant RT and also to compare between those with and without complications after adjusting for RT timing. Unless otherwise noted, a two‐sided *α *= 0.05 significance level was used.

## Results

**Figure 1:**
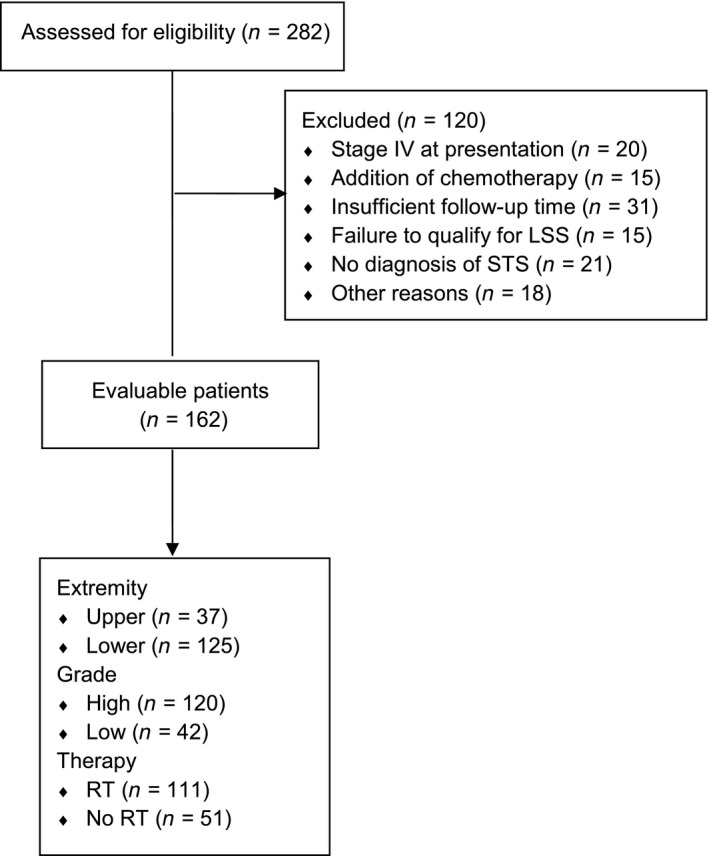
CONSORT diagram showing patient disposition.

An internal database search revealed 282 patients who were diagnosed with upper or lower extremity STS. One hundred and twenty patients (120) were excluded from the evaluable population (Fig. 1). One hundred and sixty‐two patients met inclusion criteria. Median follow‐up time for the 162 patients was 5.1 years (range: 0.8–20.3 years). RT (using 2D RT, 3D conformal radiotherapy, and IMRT techniques) was administered in 111 patients. Of those 111 patients, 36.9% received neoadjuvant RT and 63.1% received adjuvant RT. One hundred and twenty patients were G2/G3 (high‐grade) and 42 were G1 (low‐grade) STS. There were 37 upper and 125 lower extremity STSs. Table 1 details the demographic and tumor characteristics for all patients and for the two RT groups. There were slightly more females than males (51.9% vs. 48.1%) and the majority of patients were >50 years old (62.4%). Additionally, the majority of sarcomas were from lower extremities (77.2%), the right side of the body (54.9%), deep (88.9%), high‐grade (74.1%), and larger than 5 cm in diameter (65.4%). Most characteristics were similar between RT groups; however, imbalances appear in four factors. Imbalances in gender (54.1% male RT group, 35.3% male in no RT group) and tumor size categories (more tumors larger than 5 cm in the RT group as compared to the no RT group) suggest potential for confounding of the association between RT and the outcomes. The rate of close margins after LSS was higher in the RT group than the no RT group (20.7% vs. 5.9%, respectively); however, the rate of positive margins was lower in the RT group compared to the no RT group (6.3% vs. 17.7%, respectively). Additionally, there was a notable difference in distribution of tumor stages between the RT groups; patients who received RT typically had higher stage tumors (45.1% stage III in RT, 11.8% stage III in no RT). Finally, there were more high‐grade tumors in the RT group (86.5% high‐grade in RT, 47.1% high‐grade in no RT).

**Table 1:** Patient and tumor characteristics for all patients

Demographics	RT (*n* = 111)*n* (%)	No RT (*n* = 51)*n* (%)	All (*n = *162)*n* (%)
Gender			
Male	60 (54.1)	18 (35.3)	78 (48.1)
Female	51 (45.9)	33 (64.7)	84 (51.9)
Age (years)			
≤50	42 (37.8)	19 (37.3)	61 (37.6)
>50	69 (62.2)	32 (62.7)	101 (62.4)
Site			
Upper extremity	27 (24.3)	10 (19.6)	37 (22.8)
Lower extremity	84 (75.7)	41 (80.4)	125 (77.2)
Side			
Right	60 (54.1)	29 (56.9)	89 (54.9)
Left	51 (45.9)	22 (43.1)	73 (45.1)
Grade			
Low	15 (13.5)	27 (52.9)	42 (25.9)
High	96 (86.5)	24 (47.1)	120 (74.1)
Depth			
Superficial	12 (10.8)	6 (11.8)	18 (11.1)
Deep	99 (89.2)	45 (88.2)	144 (88.9)
Size (cm)			
≤5	35 (31.5)	21 (41.2)	56 (34.6)
>5	76 (68.5)	30 (58.8)	106 (65.4)
Margin			
Positive	7 (6.31)	9 (17.7)	16 (9.9)
Close	23 (20.7)	3 (5.9)	26 (16.1)
Negative	81 (73.0)	36 (70.6)	117 (72.2)
Unknown	0 (0.0)	3 (5.9)	3 (1.9)
AJCC stage			
IA	5 (4.5)	6 (11.8)	11 (6.8)
IB	12 (10.8)	19 (37.3)	31 (19.1)
IIA	19 (17.1)	11 (21.6)	30 (18.5)
IIB	23 (20.7)	5 (9.8)	28 (17.3)
III	50 (45.1)	6 (11.8)	56 (34.6)
Unknown	2 (1.8)	4 (7.8)	6 (3.7)

**Figure 2:**
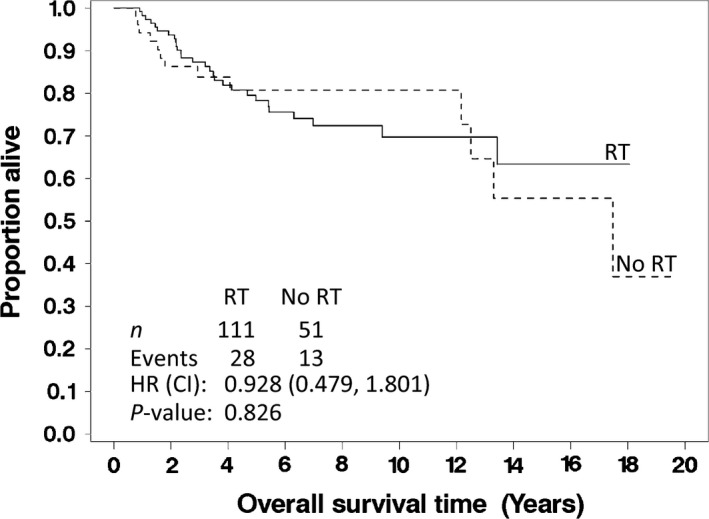
Overall survival for all patients with soft tissue sarcomas of the extremity, stratified by whether they received (solid line) or did not receive (dashed line) radiation therapy after limb‐sparing surgery. The *P*‐value listed is for the log‐rank test.

Overall, we found a trend favoring RT (but not significant at *α *= 0.05) for time to LR; however, there was no impact of RT on RFS, OS, or time to systemic recurrence. Figure 2 shows the OS distributions for the RT and no RT groups when evaluating all tumor grades.

### Patients with high‐grade sarcomas

Among the 120 patients who had high‐grade extremity STSs, 96 received RT. Median follow‐up time for high‐grade patients was 4.9 years (range: 0.8–20.3 years), which is similar to the follow‐up for the entire population. Table 2 details the patient and tumor characteristics for the RT groups among patients with high‐grade STS. Imbalances in gender (53.1% male in RT group vs. 20.8% male in no RT group), tumor size (68.7% >5 cm in RT group vs. 37.5% >5 cm in no RT group), tumor stage (52.1% stage III in RT group vs. 25.0% stage III in no RT group), margin status (19.8% close in RT group, vs. 0.0% close in no RT group), and age (64.6% older than 50 in RT group vs. 58.3% older than 50 in no RT group) between the RT groups could be potential confounders.

**Table 2:** Patient and tumor characteristics for high‐grade patients only

Demographics	RT (*n* = 96)*n* (%)	No RT (*n *=* *24)*n* (%)	All (*n *=* *120)*n* (%)
Gender			
Male	51 (53.1)	5 (20.8)	56 (46.7)
Female	45 (46.9)	19 (79.2)	64 (53.3)
Age (years)			
≤50	34 (35.4)	10 (41.7)	44 (36.7)
>50	62 (64.6)	14 (58.3)	76 (63.3)
Site			
Upper extremity	23 (24.0)	6 (25.0)	29 (24.2)
Lower extremity	73 (76.0)	18 (75.0)	91 (75.8)
Side			
Right	52 (54.2)	13 (54.2)	65 (54.2)
Left	44 (45.8)	11 (45.8)	55 (45.8)
Depth			
Superficial	11 (11.5)	2 (8.3)	13 (10.8)
Deep	85 (88.5)	22 (91.7)	107 (89.2)
Size (cm)			
≤5	30 (31.3)	15 (62.5)	45 (37.5)
>5	66 (68.7)	9 (37.5)	75 (62.5)
Margin			
Positive	5 (5.2)	5 (20.8)	10 (8.3)
Close	19 (19.8)	0 (0.0)	19 (15.8)
Negative	72 (75.0)	19 (79.2)	91 (75.8)
AJCC stage			
IA	0 (0.0)	0 (0.0)	0 (0.0)
IB	2 (2.1)	0 (0.0)	2 (1.7)
IIA	19 (19.8)	11 (45.8)	30 (25.0)
IIB	23 (24.0)	5 (20.8)	28 (23.3)
III	50 (52.1)	6 (25.0)	56 (46.7)
Unknown	2 (2.1)	2 (8.3)	4 (3.3)

**Figure 3:**
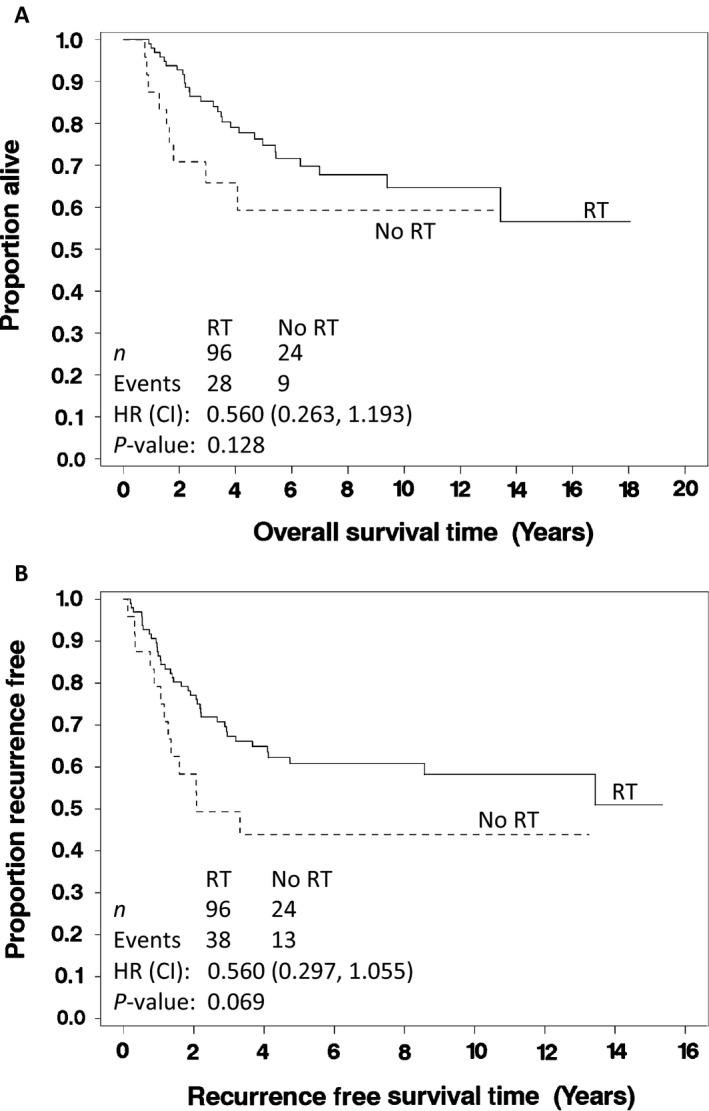
(A) Overall survival of patients and (B) overall recurrence‐free survival with high‐grade soft tissue sarcomas of the extremities, who received (solid) or did not receive (dashed) radiation therapy after limb‐sparing surgery.

**Figure 4:**
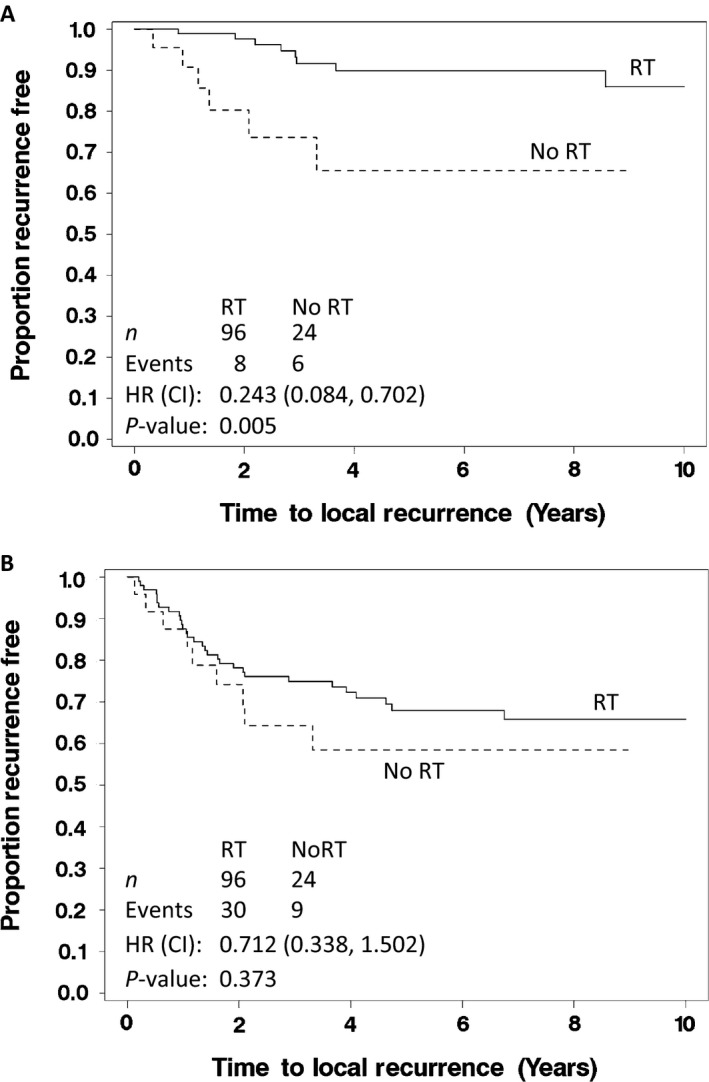
(A) Time to local recurrence in patients and (B) time to systemic recurrence with high‐grade soft tissue sarcomas of the extremities, who received (solid) or did not receive (dashed) radiation therapy after limb‐sparing surgery. The *P*‐value listed is from the log‐rank test.

Univariable results showed a trend for improved OS with RT, resulting in a 44% reduction in the risk of death in patients with high‐grade tumors (*P* = 0.128, HR: 0.560 [95% CI: 0.263, 1.193], Fig. 3A). Additionally, there was a strong trend for improved RFS associated with RT where a 44% reduction in the risk of recurrence or death was observed (*P* = 0.069, HR: 0.560 [95% CI: 0.297, 1.055], Fig. 3B). RT significantly reduced the risk of LR (*P* = 0.005, HR: 0.243 [95% CI: 0.084, 0.702], Fig. 4A). For time to systemic recurrence (Fig. 4B), although RT appeared to have a favorable impact, this difference was not statistically significant (*P* = 0.373, HR: 0.712 [95% CI: 0.338, 1.502]).

Because of the imbalances in patient and tumor characteristics noted above, adjusting for factors that could inflate or attenuate the impact of RT on survival outcomes was necessary. Tables 3, 4, 5, 6 contain the univariable and multivariable results for OS, RFS, TTR‐local, and TTR‐systemic, respectively. For each outcome, adjusting for patient and tumor characteristics increased the effect size associated with RT. Almost a 65% reduction in the risk of a recurrence or death (any cause) in patients with RT (*P* = 0.003, HR: 0.362 [95% CI: 0.187, 0.699]), after adjusting for age and tumor size was observed. Time to LR was significantly impacted by RT (HR: 0.109 [95% CI: 0.030, 0.392] *P* = 0.001,), after adjusting for tumor size and margin status. Finally, there was a trend for improved time to systemic recurrence associated with RT (*P* = 0.128, HR: 0.549, [95% CI: 0.254, 1.188]), after adjusting for tumor site and tumor size. Increases in RT effect sizes observed in the adjusted hazard ratios reflect the notable baseline imbalances in gender, age, or tumor size, all favoring the no RT group.

**Table 3:** Univariable and multivariable model results for overall survival in high‐grade patients

	Univariable model			Multivariable model		
	HR	95% CI	*P* a	HR	95% CI	*P* b
RT						
Yes vs. no	0.560	0.263–1.193	0.128	0.275	0.117–0.647	.003
Gender						
Male vs. female	1.527	0.797–2.927	0.202	2.602	1.239–5.465	.012
Age						
*>*50 years vs. ≤50 years	2.544	1.158–5.588	0.020	2.936	1.258–6.854	.013
Tumor site						
Lower vs. upper	1.244	0.568–2.724	0.585	–	–	–
Tumor side						
Right vs. left	1.152	0.601–2.207	0.671	–	–	–
Tumor size						
>5 cm vs. ≤5 cm	2.499	1.141–5.476	0.022	2.536	1.115–5.771	.027
Margin						
Close vs. positive	0.951	0.286–3.163	0.412	*–*	*–*	–
Negative vs. positive	0.604	0.209–1.744		*–*	*–*	

Base models were found for each of the endpoints through model selection procedures in order to estimate adjusted hazard ratios for the impact of RT.

a A log‐rank test was used to determine these *P*‐values.

b A Wald chi‐square test was used to determine these *P*‐values.

**Table 4:** Univariable and multivariable model results for recurrence‐free survival in high‐grade patients

	Univariable model			Multivariable model		
	HR	95% CI	*P* a	HR	95% CI	*P* b
RT						
Yes vs. no	0.560	0.297–1.055	0.069	0.362	0.187–0.699	0.003
Gender						
Male vs. female	0.992	0.572–1.719	0.976	–	–	–
Age						
>50 years vs. ≤50 years	2.083	1.107–3.920	0.023	1.765	0.923–3.375	0.086
Tumor site						
Lower vs. upper	1.461	0.732–2.918	0.283	–	–	–
Tumor side						
Right vs. left	0.983	0.567–1.704	0.952	–	–	–
Tumor size						
>5 cm vs. ≤5 cm	3.045	1.522–6.089	0.002	3.386	1.630–7.034	0.001
Margin						
Close vs. positive	0.751	0.251–2.244	0.610	–	–	–
Negative vs. positive	0.636	0.249–1.622		–	–	

Base models were found for each of the endpoints through model selection procedures in order to estimate adjusted hazard ratios for the impact of RT.

a A log‐rank test was used to determine these *P*‐values.

b A Wald chi‐square test was used to determine these *P*‐values.

**Table 5:** Univariable and multivariable model results for time to local recurrence in high‐grade patients

	Univariable model			Multivariable model		
	HR	95% CI	*P* a	HR	95% CI	*P* b
RT						
Yes vs. no	0.243	0.084–0.702	0.005	0.109	0.030–0.392	0.001
Gender						
Male vs. female	0.442	0.139–1.410	0.168	–	–	–
Age						
>50 years vs. ≤50 years	1.922	0.600–6.164	0.272	–	–	–
Tumor site						
Lower vs. upper	0.671	0.225–2.005	0.475	–	–	–
Tumor side						
Right vs. left	1.527	0.512–4.561	0.448	–	–	–
Tumor size						
*>*5 cm vs. ≤5 cm	3.012	0.837–10.842	0.092	4.413	1.161–16.789	0.029
Margin						
Close vs. positive	0.315	0.070–1.413	0.011	0.492	0.095–2.556	0.009
Negative vs. positive	0.152	0.044–0.520		0.134	0.036–0.501	

Base models were found for each of the endpoints through model selection procedures in order to estimate adjusted hazard ratios for the impact of RT.

a A log‐rank test was used to determine these *P*‐values.

b A Wald chi‐square test was used to determine these *P*‐values.

**Table 6:** Univariable and multivariable model results for time to systemic recurrence in high‐grade patients

TTR‐systemic	Univariable model			Multivariable model		
Covariate	HR	95% CI	*P* a	HR	95% CI	*P* b
RT						
Yes vs. no	0.712	0.338–1.502	0.373	0.549	0.254–1.188	0.128
Gender						
Male vs. female	0.894	0.475–1.683	0.728	*–*	*–*	*–*
Age						
>50 years vs. ≤50 years	1.473	0.746–2.909	0.265	*–*	*–*	*–*
Tumor site						
Lower vs. upper	3.191	1.134–8.982	0.028	2.534	0.889–7.225	0.082
Tumor side						
Right vs. left	1.006	0.536–1.889	0.984	*–*	*–*	*–*
Tumor size						
*>*5 cm vs. ≤5 cm	2.837	1.302–6.181	0.009	2.857	1.271–6.422	0.011
Margin			0.838			
Close vs. positive	0.827	0.197–3.461	0.795	*–*	*–*	*–*
Negative vs. positive	1.098	0.335–3.593	0.878	*–*	*–*	*–*

Base models were found for each of the endpoints through model selection procedures in order to estimate adjusted hazard ratios for the impact of RT.

a A log‐rank test was used to determine these *P*‐values.

b A Wald chi‐square test was used to determine these *P*‐values.

Local RFS was significantly improved with RT in both univariable (*P* = 0.005) and multivariable (*P* = 0.001) analyses. Results for systemic RFS reflected those observed for OS, where the univariable results showed a trend (*P* = 0.134) while the multivariable results showed significant improvement for RT (*P* = 0.013).

### Patients with low‐grade sarcomas

Of the 42 patients with low‐grade sarcomas, 15 received RT. Median follow‐up time was 7.1 years (range: 2.0–19.6 years) for low‐grade patients, which was somewhat longer than the follow‐up time for the high‐grade population. This was mainly associated with a much longer median time to death for those who died in the low‐grade population (12.9 vs. 2.4 years), whereas the median follow‐up time for censored patients was very similar between the low‐grade and high‐grade populations (6.5 vs. 6.4 years, respectively). Only four low‐grade patients died during follow‐up; no deaths were observed in patients who received RT. Additionally, only two LRs occurred (one in each the RT and no RT groups). No systemic recurrences were observed in any patients. This small number of events did not support detailed individual analyses of patients with low‐grade STS.

### Patient complications

Overall, patients who received RT were significantly (*P* = 0.037) more likely to experience a major complication (16.2%) than those who did not receive RT (3.9%). However, in high‐grade sarcomas only, the difference in complication rate was not significant (16.7% vs. 8.3%, *P* = 0.522). The majority of complications overall and among patients with high‐grade tumors were pathologic fractures (72.2% and 68.8%, respectively) (Tables 7 and 8). Only two patients with low‐grade sarcomas experienced major complications, both of which were in the RT group (13.3% vs. 0%, *P* = 0.122).

Mean radiation dose was 57 Gy (median 60 Gy). Patients with high‐grade STS received a median dose of 60 Gy and patients with low‐grade STS received a median dose of 61.2 Gy (*P* = 0.852). For neoadjuvant versus adjuvant RT, the mean doses were 50.7 Gy (median 50 Gy) and 60.7 Gy (median 63 Gy), respectively, which is in alignment with our institutional standards. Adjuvant radiation doses were numerically different (although not significantly different) among margin status groups (positive: 64.6 Gy, close: 61.0 Gy, negative: 60.3 Gy, *P* = 0.415). Patients who experienced a fracture, or any complication, did not experience higher radiation doses than those who did not, after adjusting for neoadjuvant versus adjuvant timing of RT (56.1 vs. 55.7 Gy, *P* = 0.766; 56.3 vs. 55.6 Gy, *P* = 0.630, respectively).

**Table 7:** Incidence of major complications in all patients between therapy groups

Complication	RT (*n* = 111)		No RT (*n* = 51)		*P* a
	No.	%	No.	%	
Amputation	6	5.4	2	3.9	>0.999
Pathologic fracture	13	11.7	0	0.0	0.010
Radiation‐induced sarcoma	3	2.7	0	0.0	0.552
Any complication	18	16.2	2	3.9	0.037

a A two‐sided Fisher's exact test was used to determine these *P*‐values.

**Table 8:** Incidence of major complications in high‐grade patients between therapy groups

Complication	RT (*n* = 96)		No RT (*n* = 24)		*P* a
	No.	%	No.	%	
Amputation	5	5.2	2	8.3	0.6260
Pathologic fracture	11	11.5	0	0.0	0.1181
Radiation‐induced sarcoma	2	2.1	0	0.0	>0.999
Any complication	16	16.7	2	8.3	0.5224

a A two‐sided Fisher's exact test was used to determine these *P*‐values.

## Discussion

The results of this study demonstrate a significant improvement in overall survival, recurrence‐free survival, and time to local recurrence with RT among high‐grade soft tissue sarcoma patients. While the small number of events (2 local recurrences, 0 systemic recurrences, and 4 deaths) limited definitive analyses of the low‐grade cohort, the attenuated results for the overall populations would indirectly suggest limited benefit of RT in low‐grade patients.

In multivariable analyses adjusting for age, gender, and tumor size, we demonstrated an over 70% reduced risk of death high‐grade patients who were treated with RT. A retrospective SEER analysis published by Koshy et al. also reported an improvement in OS in this patient population using multivariable analyses (HR = 0.67, 95% CI: 0.57–0.79) 18. After adjusting for age and tumor size, we found an almost 65% reduction in the risk of recurrence or death from any cause with RT. In a study published by Yang et al. in 1998, patients who received adjuvant RT had improvement in local RFS (*P* = 0.003), but no difference in OS (*P* = 0.71) 10. In a follow‐up analysis (median = 17.9 years), LR was improved in patients who received RT compared to patients who only received LSS (25% vs. 1.4%, *P* = 0.0001), but with higher incidence of wound complications, clinically significant edema, and functional limb deficits. OS numerically favored the addition of RT but was again not significant (*P* = 0.22) 7. Additionally, in this study, time to local recurrence was improved for patients who received RT. Improvement in LR by RT after LSS has been documented in several other studies 10, 15.

We observed more major complications, in patients (all grades) who received RT compared to those who did not receive RT (16.2% of 111 vs. 3.9% of 51 patients, *P* = 0.037). The rate of major complications in all patient receiving RT was similar to a recent report by Roeder et al.^20^. However, despite a doubling in complication rate in patients with high‐grade sarcomas (16.7% of 96 vs. 8.3% of 24 patients), this difference did not approach statistical significance (*P* = 0.522). As such, there appears to be a positive benefit–risk ratio associated with RT in patients with high‐grade sarcomas.

Pathologic fracture was found as the most common radiation‐associated complication. The rate of pathologic fracture for patients treated with RT in this study was 11.7% in all patients and 11.5% in high‐grade patients. This rate is similar to that reported by Beane et al. (10%) but almost double to that reported by McGee et al. and Roeder et al. 7, 20, 21. RT and associated complications have been found to be an independent predictor of poor patient disability with standardized outcome scores 22. Radiation‐induced STS places patients at a further increased risk for local and systemic recurrence compared to sporadic primary sarcoma 23. However, we observed a low rate of radiation‐induced STS (2.1% in high‐grade STS). Efficacy of RT in patients with high‐grade tumors should be discussed in the context of the potential for these additional complications.

O'Sullivan et al. examined the effect of modern radiation techniques, specifically image‐guided intensity‐modulated radiation therapy (IG‐IMRT), on pathologic fractures in 59 patients treated in the neoadjuvant setting. While 30.5% developed acute wound complications, no patients suffered bone fracture 24. Another study of preoperative image‐guided radiation therapy (IGRT) to a reduced target volume found a low rate of grade 2 + toxicity at 2 years (10.5%) 25 and a comparison of external beam radiation therapy and IMRT found IMRT improved LR (HR = 0.46, 95% CI: 0.24–0.89; *P* = .02)^4^. Modern RT techniques may improve the complication and LR rates experienced in patients.

A main limitation of this study, as with almost all retrospective cohort studies, is that selection bias cannot be ruled out. Treatment was determined by a multidisciplinary sarcoma tumor board. If the cohort of subjects who received treatment with RT differed favorably from the cohort who did not receive RT, then selection bias could have occurred. We noted imbalances in gender and tumor characteristics between RT treatment groups, and therefore attempted to minimize confounding by controlling for these factors using multivariable analyses, a common approach in retrospective cohort studies. Additionally, because of the retrospective nature of this study, it is possible that there are other confounders that we were not able to incorporate in the multivariable analyses (e.g., comorbidities). Specifically, related to the use of radiation therapy, we did not have information available on radiation field margin radiation technique (2D RT, 3D conformal radiotherapy, and IMRT), fields, or radiation margin status at the patient level. Unfortunately, because of this, we are unable to make inferences on the effect of modern RT techniques, such as IMRT, on complications, such as fractures. Future research could address this limitation.

Despite these limitations, a major strength of this study is that STS patients were treated in the community‐based, nonacademic setting. Thus, our results are likely generalizable to patients treated at other community‐based centers. Another strength of this study is the relatively large sample size in this setting. Lastly, literature on the impact of RT on recurrence, survival outcomes (particularly OS), and complications is limited for STSs of the extremity, thus our study contributes to the gap in knowledge on these topics.

Our 18‐year community‐based experience of patients with extremity STSs demonstrates significant improvement in LR, RFS, and OS, in patients with high‐grade tumors receiving RT. We report increased incidence of major complications with RT (although not significantly among high‐grade patients), which can impact quality of life. We recommend considering external beam RT in patients with resected high‐grade sarcoma due to improved LR, RFS, and OS. Considering the differences in results between the entire cohort and the cohort of patients with high‐grade STS, the authors currently restrict recommendations for external beam RT with resected low‐grade STS; however, RT recommendations should be made in a multidisciplinary setting, as indications for RT may vary from patient to patient based upon American Joint Committee on Cancer (AJCC) stage and other patient‐specific factors, such as tumor location and medical comorbidities. Low LR rates with prospective discussion of RT at multidisciplinary tumor board have been reported 26 reinforcing the benefit of utilizing a multidisciplinary team.

## Conflict of Interest

There are no financial disclosures or conflicts of interest.
